# Accuracy Evaluation of 3D Pose Reconstruction Algorithms Through Stereo Camera Information Fusion for Physical Exercises with MediaPipe Pose

**DOI:** 10.3390/s24237772

**Published:** 2024-12-04

**Authors:** Sebastian Dill, Arjang Ahmadi, Martin Grimmer, Dennis Haufe, Maurice Rohr, Yanhua Zhao, Maziar Sharbafi, Christoph Hoog Antink

**Affiliations:** 1 KIS*MED (AI Systems in Medicine), Technische Universität Darmstadt, 64283 Darmstadt, Germanyzhao@kismed.tu-darmstadt.de (Y.Z.); hoogantink@kismed.tu-darmstadt.de (C.H.A.); 2 Lauflabor (Locomotion Laboratory), Centre for Cognitive Science, Technische Universität Darmstadt, 64289 Darmstadt, Germanymartin.grimmer@tu-darmstadt.de (M.G.);

**Keywords:** computer vision, human pose estimation, information fusion, MediaPipe Pose

## Abstract

In recent years, significant research has been conducted on video-based human pose estimation (HPE). While monocular two-dimensional (2D) HPE has been shown to achieve high performance, monocular three-dimensional (3D) HPE poses a more challenging problem. However, since human motion happens in a 3D space, 3D HPE offers a more accurate representation of the human, granting increased usability for complex tasks like analysis of physical exercise. We propose a method based on MediaPipe Pose, 2D HPE on stereo cameras and a fusion algorithm without prior stereo calibration to reconstruct 3D poses, combining the advantages of high accuracy in 2D HPE with the increased usability of 3D coordinates. We evaluate this method on a self-recorded database focused on physical exercise to research what accuracy can be achieved and whether this accuracy is sufficient to recognize errors in exercise performance. We find that our method achieves significantly improved performance compared to monocular 3D HPE (median RMSE of 30.1 compared to 56.3, *p*-value below $10^{-6}$) and can show that the performance is sufficient for error recognition.

## 1. Introduction

Physical therapy plays a vital role in treating a wide range of injuries and conditions, including rare disorders such as hemophilia, where physiotherapy and rehabilitation are essential for preventing disabilities and maintaining patient autonomy [1]. Ideally, therapeutic exercises are performed under the guidance of medical professionals or sports experts who can provide personalized and immediate feedback. However, many individuals lack the resources for regular supervised training sessions and instead perform the majority of their therapeutic exercises at home. Similarly, recent years have seen a spike in at-home sports exercises, with the COVID-19 pandemic serving as a catalyst [2]. Research indicates that home exercises can effectively support the recovery process after injuries [3], even without expert supervision. On the other hand, improper execution, overestimating one’s fitness level, or overexertion can lead to inefficient training or, worse, severe injuries [4]. To address these challenges, an automated evaluation system can be employed to assess exercise quality, reducing the reliance on human supervision. Due to its ease of use, low price, high level of comfort, and recent advances in computer vision, video-based human pose estimation (HPE) and motion capture (MoCap) has emerged as a relevant tool for accessible exercise supervision [5]. Another benefit is the fact that there are huge databases that feature videos of exercises freely available online, that can be used when creating and training a video-based exercise evaluation system [6].

Two-dimensional (2D) monocular HPE, i.e., the task of locating the human pose of a single person and their distinct joints in an image plane, has been shown to achieve high performance. Popular 2D approaches using deep learning techniques include OpenPose [7], DeepPose [8] and MediaPipe Pose [9]. The feasibility of popular 2D approaches has been shown both for particular medical applications like gait analysis [10] and sports activities like yoga [11]. Conversely, three-dimensional (3D) monocular HPE, i.e., the task of locating the human pose of a single person and their distinct joints in a 3D coordinate system, is more challenging than its 2D counterpart, because 3D pose estimation from monocular inputs presents an ill-posed problem, as multiple 3D predictions can correspond to the same 2D projection. Still, for obvious reasons, 3D approaches offer a more accurate representation of the human’s pose, and have been used for more complex medical applications such as joint load prediction [12]. One particularly popular 3D HPE library is MediaPipe Pose [13], which is based on the BlazePose model [9] due to its higher computational efficiency and ease of use compared to other methods, and the fact that it is open-source. While MediaPipe Pose offers 3D pose estimations from a single camera view, the z-Axis, which is oriented perpendicularly to the image plane, suffers from high noise. This deteriorates the overall estimation quality, as shown in one of our previous papers [14], where we have evaluated the accuracy of MediaPipe-based pose estimation for physical therapy.

The idea to combine the advantages of 3D pose estimation with the easier problem of 2D pose estimation has led to research focusing on multi-view pose estimation [15,16,17,18,19]. Notable recent studies include the works of Wang et al. [20], who performed 3D HPE based on a multi-scale orthogonal projection fusion network and achieved improved accuracy and robustness, and Cai et al. [21], who developed a camera-parameter-free method fusing both multi-view and multi-frame features in an approach called FusionFormer that achieved state-of-the-art HPE performance. Chen et al. [22] fused their multi-view approach with additional limb orientation data acquired through wearable inertial measurement units (IMUs), also achieving improved performance.

In theory, two sets of 2D pose coordinates are enough to reconstruct a 3D pose utilizing intrinsic and extrinsic camera parameters as well as direct triangulation to identify matching epipolar lines between the two views. However, this 3D reconstruction is still highly dependent on the quality of the 2D estimation, which is hindered by problems such as (self-)occlusion and camera angle dependency [23]. Therefore, these approaches usually scale in accuracy with the number of cameras. However, with the recent increase in estimation accuracy in 2D estimators, we were able to show in a previous work [24] that 3D stereo reconstruction, i.e., finding the position of a point in space given its position in just two images, holds up to the multi-view version. We evaluated our approach on the Panoptic database [25] of Carnegie Mellon University (CMU) and found an optimal angle between the two cameras used for reconstruction to be around 90°. While there is notably less research being conducted on stereo-view HPE, there is another recent study by Sheng et al. [26], who matched stereo-camera 2D human pose data extracted with OpenPose through a convolutional network, attaining reliable and effective 3D HPE, while highlighting the lower computational requirements of their approach compared to traditional systems. To the best of our knowledge, there is no stereo-view HPE system evaluated for accuracy in exercise assessment.

In this work, we therefore aim to verify our previous results on our own dataset of physical therapy and compare it to MediaPipe’s monocular 3D output as well as less sophisticated fusion methods, with and without prior filtering. We evaluate both the absolute positional error of the landmarks as well as task-related metrics, namely selected joint angles.

## 2. Methods

**Figure 1:**
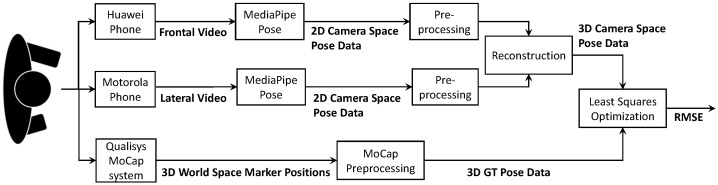
A flowchart depicting the approach to evaluate the 3D pose reconstruction through stereo camera information fusion by performed a least squares optimization to fit the reconstructed 3D pose data to the GT data.

The goal of this work is to give a quantitative evaluation of the 3D reconstruction based on stereo camera MediaPipe pose estimation with the intended purpose of physical exercises. We want to evaluate both absolute positional error as well as suitability for the specific task of recognizing wrong exercise performance. For this, we recorded our own dataset, consisting of nine subjects, performing squats, both in correct and incorrect variations. The subjects were recorded by two video cameras as well as a motion capture system. We feed the image streams of both cameras into two distinct instances of the MediaPipe Pose framework [9] to receive two sets of 2D human pose joint estimations. With methods of epipolar geometry and triangulation, we give an estimate for the 3D world coordinates and compare it to the 3D coordinates of the motion capture system. The overall approach can be seen in Figure 1.

### 2.1. Dataset

**Figure 2:**
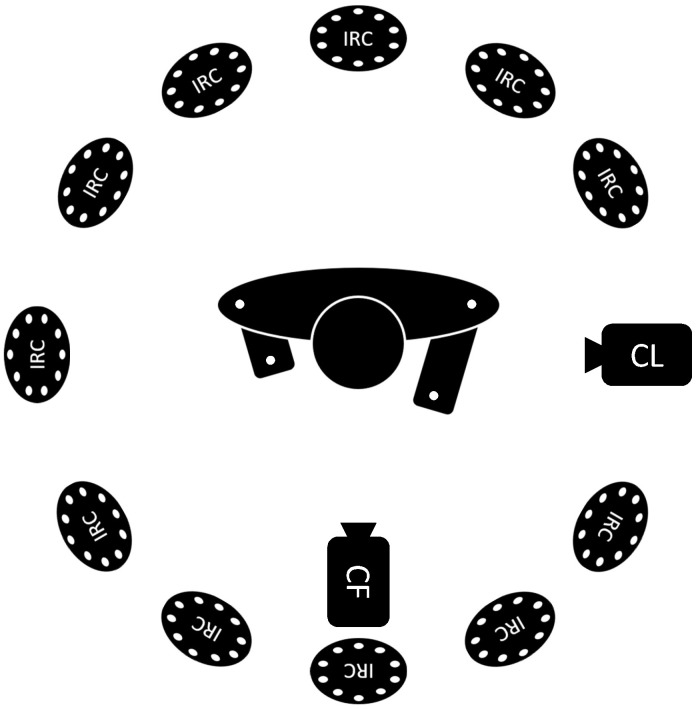
Schematic of the experimental setup. The test subject is wearing markers represented by white dots. The exact marker positions on the subject can bee seen in Figure 3a. The subject is recorded by 11 active infrared cameras (IRCs) for the MoCap system as well as a frontal RGB camera (CF) and lateral RGB camera (CL).

The dataset was recorded in January 2024 at Locomotion Laboratory of TU Darmstadt in a big meeting room with uniform lighting from the ceiling. A schematic of the complete experimental setup can be seen in Figure 2. Twelve subjects performed three different experiments, each consisting of three trials of ten repetitions of a squat, with a 2-min break in between each trial. In experiment one (E1), the squat was performed correctly, as defined by the following criteria:

- Shoulder-width stance with parallel or slightly V-shaped feet.
- Heels remain on the floor throughout the exercise.
- The spine remains straight throughout the exercise.
- Both legs are loaded symmetrically, i.e., the center of the body should not deviate from the center of the feet.

In the other experiments, the exercise was performed incorrectly. In the second experiment (E2), the subject bends forwards excessively during the squat, which also leads to a rounding of the back in some test subjects. In the third experiment (E3), the subject shifts their weight heavily to the right side, which leads to asymmetry.

The subjects were recorded by the red–green–blue (RGB) cameras of two commercially available smartphones (Motorola moto e40, Huawei Mate 20) in portrait format (1080×1920 pixels and at 30 frames per second (fps)). Each trial is captured as an individual recording. Since our previous research [24] found the angle between two cameras for 3D pose reconstruction to be optimal when it approaches 90°, the two smartphones were positioned frontal and lateral to the subjects. The phones were placed upon tripods at a height of approximately 130 cm and were positioned perpendicular to the ground.

**Figure 3:**
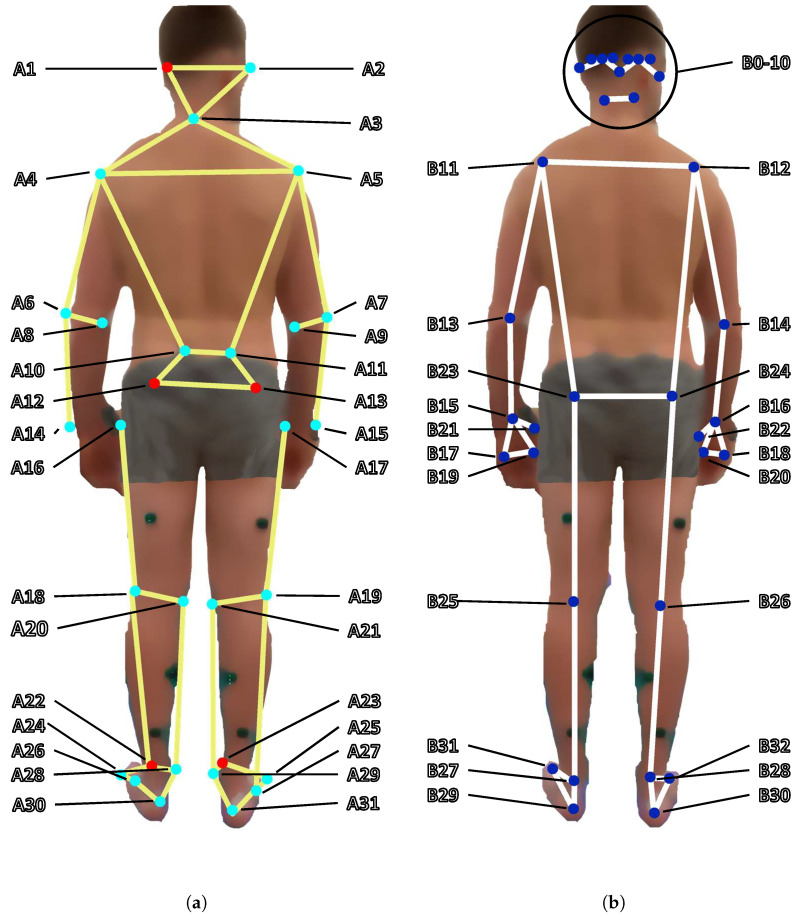
General visualization of the 31 marker positions of the MoCap system (left) and the MediaPipe Pose output (right). (**a**) Not all markers are visible from the back of the person. The ones positioned at the subject’s front are color-coded in red. In this work, we only use markers A19 and A21 relating to the right knee, A18 and A20 relating to the left knee, A27 and A29 relating to the right ankle, A26 and A28 relating to the left ankle, A13 and A17 relating to the right hip, A12 and A16 relating to the left hip, A5 for the right shoulder, A4 for the left shoulder, A7 and A9 relating to the right elbow, A6 and A8 relating to the left elbow, A15 for the right wrist and A14 for the left wrist. Explanations for the marker labels can be found in Table A1. (**b**) MediaPipe’s output consists of x-y-z coordinates of 33 different landmarks. The coordinate system’s origin is in the upper left corner of the image, with x increasing from left to right and y increasing from top to bottom. The z axis is pointed perpendicularly away from the image plane. In this work, we only consider joints B11 to B16 for the upper body and B23 to B28 for the lower body. Explanations for the joint labels can be found in Table A2.

To provide a ground truth (GT) to compare our estimate of 3D joint positions to, the subjects were also recorded by a marker-based MoCap system. The System consisted of 31 reflective markers that were taped to the subject at distinct positions and were monitored by 11 Qualisys cameras (Oqus 300+ and 310+) recording at 100 fps. The marker positions can be seen in Figure 3a. To position the markers as accurately as possible, the subjects performed all experiments only wearing shorts. The trajectories of the markers captured by the Qualisys system are assigned to their corresponding labels of the predefined marker set. Trajectory gaps below 100 samples were interpolated using the gap-filling tool of the MoCap data collection software version 2024.2 (Qualisys Track Manager). By combining the recordings from all eleven Qualisys cameras, the exact 3D position of each marker can be triangulated with sub-millimeter precision. From these reconstructed 3D MoCap marker positions, we constructed GT joint positions that were used as GT for this work.

Due to problems with the MoCap system, three subjects (S1, S2 and S5) had to be excluded from the measurement, leaving a total of 81 individual recordings with 810 instances of squats over nine subjects.

### 2.2. MediaPipe Pose

We present a brief overview of the BlazePose model, which is utilized by MediaPipe and therefore serves as the baseline for our research. MediaPipe’s output consists of x-y-z-coordinates of 33 different joint positions as well as a visibility estimate for each joint, ranging from 0 to 1, with a higher value indicating more confidence on the estimated joint position. The coordinate system in which these positions are given depends on the operating mode. In *landmark* mode, the output coordinates are given as image coordinates, where the x-y-plane is parallel to the image plane and the z-axis is oriented perpendicularly away from the camera. In *worldmark* mode, these image coordinates are mapped onto an internal model of a human to create an estimate for real-world coordinates in meters. This coordinate system is centered between the hip joints and moves with the subject. We only consider *landmark* mode for this work. A visualization of the landmarks is given in Figure 3b.

As can be seen in Figure 3, the 33 joint positions output by MediaPipe and the 31 markers tracked by the MoCap system do not match up one-to-one. Therefore, we define a common subset of 12 joints: shoulder, elbow, wrist, hip, knee, and ankle for both the left and right side. Since the MoCap marker positions are always at the subject’s surface while the joint positions output by MediaPipe lie within the actual joint, we have to perform preprocessing to make the positions comparable. For example, for the left elbow, we take the mean position between the markers A6 and A8, which we set equivalent to joint B13 from the MediaPipe output. Similarly, for the left hip, we create a fictional MoCap marker from the x-coordinate of A12, the y-coordinate of A16 and the z-coordinate from A16 and set it equivalent to B23 from the MediaPipe output. A full set of equivalent marker and joint position assignments is given in Table 1.

MediaPipe also outputs a direct estimate for joints’ depth. This z-coordinate estimation is different than the estimation of the x- and y-coordinates, as it is not located within the image plane. It is therefore less reliable, as 3D pose estimation from monocular inputs presents an ill-posed problem, as multiple 3D predictions can correspond to the same 2D projection. MediaPipe addresses this issue by fitting their 2D projection to an internal model of a human with an assumed standard height. The best fit is selected for depth estimation. We compare the 3D coordinates provided from MediaPipe both on the frontal and the lateral camera to our own approach. Since the MediaPipe output does contain noise, we compare all methods in their unfiltered version with a version where the signals have been filtered. To find an optimal filter, a moving-average filter, a Butterworth low-pass filter and a Savitzky–Golay filter were tried out. Their parameters were optimized through a grid-search.

### 2.3. Three-Dimensional Pose Reconstruction

To give more context to the relationship between the different 2D and 3D coordinate systems, the following section introduces definitions and fundamentals of coordinate systems and 3D pose reconstruction. In this work, we will use homogeneous coordinates, i.e., coordinates with an extra scaling dimension, which helps display transformations between coordinate systems in a mathematically concise way. To reduce complexity in notation, homogeneous coordinates will be denoted by a tilde symbol.

We define four different coordinate systems: 2D image coordinates x=[x,y], 3D image coordinates $x_{3D}=\left[xz,yz,z\right]$, 3D camera coordinates $p_{\mathrm{cam}}=\left[x_{\mathrm{cam}},y_{\mathrm{cam}},z_{\mathrm{cam}}\right]$ and 3D world coordinates $p_{\mathrm{world}}=\left[x_{\mathrm{world}},y_{\mathrm{world}},z_{\mathrm{world}}\right]$. In the following, we will briefly describe how these four coordinate systems relate to each other.

The 3D camera coordinate system is defined such that the camera is positioned at the origin of the Euclidean coordinate system, with the camera’s principal axis pointing directly along the z-axis. The image coordinate space is related to the 3D camera coordinate system through a projection, often approximated by the pinhole camera model. The pinhole camera model provides a fundamental explanation of how images are created through projection. It illustrates the geometric relationship between objects in space and their images on a flat image plane [27]. When applying the pinhole camera model, the projection from 3D camera coordinates $p_{\mathrm{cam}}$ into image space image coordinates $x_{3D}$ is given by

(1) $$x_{3D}=Kp_{\mathrm{cam}},$$

where K is referred to as the camera calibration matrix, which includes the intrinsic parameters of the camera. From the definition of x and $x_{3D}$, it directly follows that $\tilde{x}=\frac{1}{z}x_{3D}$.

The assumptions of the pinhole camera model are idealized, and in real cameras, lenses and other optical elements are used to further focus and direct light. The curvature and material of the lens cause light rays not to be perfectly focused on a point, leading to both radial and tangential distortions in the image. Overall, we consider the following distortion coefficients d:

$$d=\left(k_{1},k_{2},p_{1},p_{2},k_{3}\right)$$

Both the camera calibration matrix and the distortion coefficients can easily be estimated through sample images of a well-defined pattern, usually a chess board. We use them to undistort the 2D image coordinates x we receive from MediaPipe and then calculate the so-called normalized image coordinates:

(2) $${\tilde{x}}_{\mathrm{normalized}}=K^{-1}\tilde{x}=\frac{1}{z_{\mathrm{cam}}}p_{\mathrm{cam}},$$

which are independent of the camera-specific values for resolution and focal length. This transformation has to be performed only once and the corresponding camera-specific parameters can be ignored for the following sections. From now on, all mentions of the image coordinates refer to the normalized image coordinates.

The relationship between the 3D world coordinate system and the 3D camera coordinate system is described by the camera rotation and translation. Given a point $p_{\mathrm{world}}$ with coordinates $(x_{\mathrm{world}},y_{\mathrm{world}},z_{\mathrm{world})}$ in the world coordinate system, to transform this point into the camera coordinate system, we must account for the camera’s position and orientation in the world coordinate system. The camera is located at a specific point c in the world coordinate system, given by the vector $c=(c_{x},c_{y},c_{z})$. The orientation of the camera in space is described by a rotation matrix $R\in R^{3\times 3}$, which transforms points from the world coordinate system into the camera coordinate system, taking into account the orientation of the camera.

The overall transformation is then given by $p_{\mathrm{cam}}=R\left(p_{\mathrm{world}}-c\right)$. In homogeneous coordinates, the translation can be expressed by a matrix multiplication, resulting in the following equation:

(3) $${\tilde{p}}_{\mathrm{cam}}=\left[\begin{array}{cc} R & -Rc\\ 0 & 1 \end{array}\right]{\tilde{p}}_{\mathrm{world}}=P_{\mathrm{world},\mathrm{cam}}{\tilde{p}}_{\mathrm{world}},$$

where −Rc is usually referred to as the translation vector t and $P_{\mathrm{world},\mathrm{cam}}\in R^{4\times 4}$ is called the projection matrix from world to camera coordinates.

If we have not one but two cameras c1 and c2, the two 3D camera coordinate systems relate to one another through a rotation and translation, which can be expressed by

(4) $$p_{c1}=R_{21}p_{c2}+t_{21},$$

where $R_{21}\in R^{3\times 3}$ and $t_{21}\in R^{3\times 1}$ denote the rotation and translation from camera coordinate system 2 to camera coordinate system 1, respectively. The relationship between the two coordinate systems can therefore be defined as

(5) $${\tilde{x}}_{c1}E_{21}{\tilde{x}}_{c2}=0,$$

where the so-called essential matrix $E_{21}=t_{21}\times R_{21},\;E_{21}\in R^{3\times 3}$ describes the Euclidean transformation from camera coordinate system 2 to 1. This equation enables us to calculate the relationship between the two cameras directly from a set of image points that form a linear system of equations. In the absence of noise, this problem is trivial and can be solved with a minimum of seven corresponding points. When Gaussian noise is present, the problem may be formulated as a least-squares minimization problem [28], which we solve with the LMS (least median of squares) algorithm [29] to accommodate for outliers. The retrieved essential matrix can be decomposed with the singular value decomposition to obtain an estimate for $R_{21}$ and $t_{21}$ [30].

The resulting problem of finding an optimal estimation for 3D camera coordinates from two corresponding sets of 2D cameras can again be formulated as a linear system of equations, which we solve with Direct Linear Transform (DLT) and SVD [31].

Since the cameras in the experiment were positioned approximately perpendicular to one another, the rotation from the lateral camera to the frontal camera, as given in Equation (4), is approximately given as

(6) $$R_{lr}=\left[\begin{array}{ccc} 0 & 0 & 1\\ 0 & 1 & 0\\ -1 & 0 & 0 \end{array}\right].$$

With that, we can also combine the coordinates output by MediaPipe’s 2D image coordinate estimation directly by using the x-coordinate of the lateral camera as the z-coordinate of the frontal camera and the x-coordinate of the frontal camera as the negative z-coordinate of the lateral camera. We use this simple orthogonal combination of the two sets of coordinates as a reference method to compare our approach of epipolar geometry against.

### 2.4. Evaluation Metrics

Overall, we evaluate four different methods: the main approach of 3D reconstruction through epipolar geometry, the simple approach of 3D reconstruction through orthogonal combination, as well as the monocular 3D MediaPipe outputs from the frontal and lateral camera. Since each of these has its own coordinate system, we transform their coordinates into the original GT coordinate system through a least-squares optimization to achieve maximum comparability. The residuals of this final transformation are used as a metric of absolute accuracy to evaluate how well this 3D estimation method works. These residuals can be interpreted as a mean root-mean-square error (RMSE) over all joints and are comparable to a common metric called Mean Per Joint Position Error (MPJPE). The RMSE is evaluated for statistical significance with a *t*-test and a significance level of α=0.05. Since we want to evaluate both the absolute accuracy of the approach as well as its suitability for the task of error recognition in physical therapy, we also calculate the RMSE of the hip and knee angles, which are relevant for the squat exercise. The hip angle is defined as the 3D angle spanned by the shoulder joint, hip joint and knee joint. Similarly, the knee angle is defined as the 3D angle spanned by the hip joint, knee joint and ankle joint. Both are calculated for the left and right side of the subjects, respectively.

## 3. Results and Discussion

In this section, we will present the results of the reconstruction. First, we want to evaluate the absolute accuracy of the reconstruction, as represented by the RMSE of the transformation of the reconstructed 3D pose data to the GT coordinate system, as described in Section 2.4. We will compare all methods as well as investigate how well the reconstruction worked for the different subjects and experiments. Afterwards, we will look at the absolute error of knee and hip angles, to see if the reconstruction helps with solving the exercise-specific task of classifying the different experiments.

In our previous work [24], we achieved a global minimum of 25.4. However, the results are not perfectly comparable due to slight variations in the marker positions of the ground truth. Furthermore, since the marker positions of the GT and MediaPipe joint positions are not perfectly equivalent, we expect a minimum value for the RMSE that we cannot surpass.

### 3.1. Accuracy

**Figure 4:**
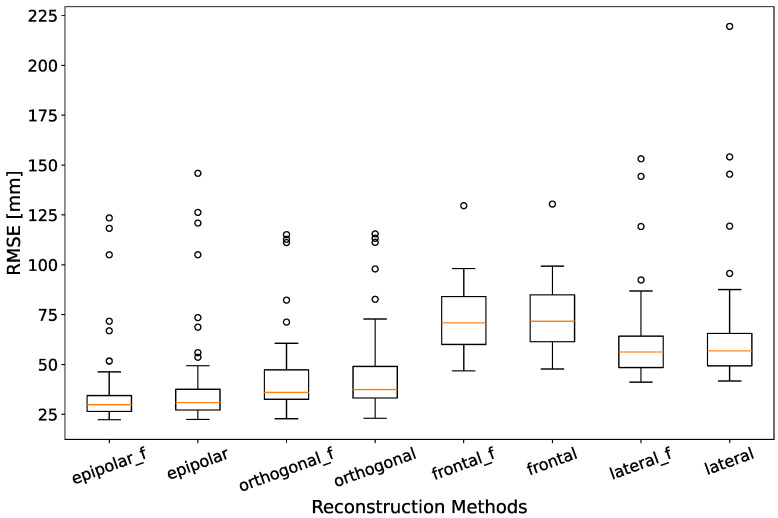
RMSE over all 81 recordings, for all reconstruction methods. The suffix _f denotes whether the signals were filtered by a 4th-order Butterworth low-pass with cut-off frequency of 2 Hz before fusion. All other filter methods performed worse and were therefore excluded from the graph. For visual clarity, three singular outliers above 225 were excluded from the graph.

Figure 4 shows the RMSE of the fitting of the reconstructed 3D pose data to the GT coordinate system for all reconstruction methods. Each individual boxplot contains all 81 videos, independent of the specific subject and experiment. As can be seen, our proposed method of epipolar reconstruction achieves the lowest median RMSE, with 30.9 mm and 30.1 mm for the unfiltered and filtered signal, respectively. The differences between the epipolar method and the single-camera approaches is significant (all *p*-values below $10^{-6}$). However, the difference between the epipolar method and the orthogonal method is not significant (*p*-values of 0.057 and 0.120 for the unfiltered and filtered signal, respectively). We can also see that while the low-pass filter, intended to smooth out noise, decreases the median RMSE for all reconstruction methods, the improvement is not significant (p=0.997). All other filter methods had a comparable but slightly worse performance. This might be because the MediaPipe library already uses an internal Kalman filter to smooth the signals, making additional filtering redundant. Furthermore, some filter methods like the moving average filter suffer from the fact that the squat movement features two sudden shifts in direction, when the subject reaches the highest and lowest point of the movement, leading to a visible delay in the filtered signal. Since no filter method led to a significant improvement in performance, we only consider the unfiltered methods in the following. The filtered epipolar method contains a single outlier video, where the RMSE reaches its overall maximum of 292.5. For visual clarity, this outlier as well as two other outliers for the other methods were excluded from the figure.

We can compare these results to previously mentioned related literature. The multi-person multi-view fusion approach by Joo et al. [25] presented with the Panoptic database achieved average errors between 39.4 mm and 62.5 mm, depending on the subject and task. The multi-view approach of Wang et al. [20] was also evaluated on the Panoptic database, achieving MPJPE values as low as 17.57 mm. Cai et al. [21] evaluated their approach on the Human3.6M [32] and TotalCapture [33] datasets for different numbers of views, achieving MPJPE values of 30.8 mm when using a stereo-view setup and 25.3 mm when using a setup with four views. Chen et al. [22] were able to reach an MPJPE of as low as 22.7 on the TotalCapture dataset with their approach of fusing a multi-view setup with IMU data. The binocular approach of Sheng et al. [26] reached RMSE values between 27.2 mm and 44.2 mm on their own dataset, depending on the action performed by the subject. In comparison, we can see the limitation of our stereo-view approach, as it performs worse than most multi-view approaches. Besides just having more available information, these approaches also have the benefit of being less susceptible to an erroneous detection in a single camera view. However, our approach outperforms the other stereo-view approaches. Furthermore, it has to be noted that none of the datasets, on which the literature approaches were evaluated, featured explicit footage of physical exercise.

We can also see that on our data, the single-camera approaches based on the frontal or lateral camera perform the worst. This is to be expected since, as explained before, 3D estimation from a single view is an ill-posed problem. We can also observe that the 3D estimation based on the frontal camera yields worse results than the one based on the lateral camera. One possible explanation for that is the high left–right symmetry of the performed squats. Therefore, the self-occlusion of one half of the human pose when looking from a lateral perspective is of lower importance than the missing depth information from the frontal view.

**Figure 5:**
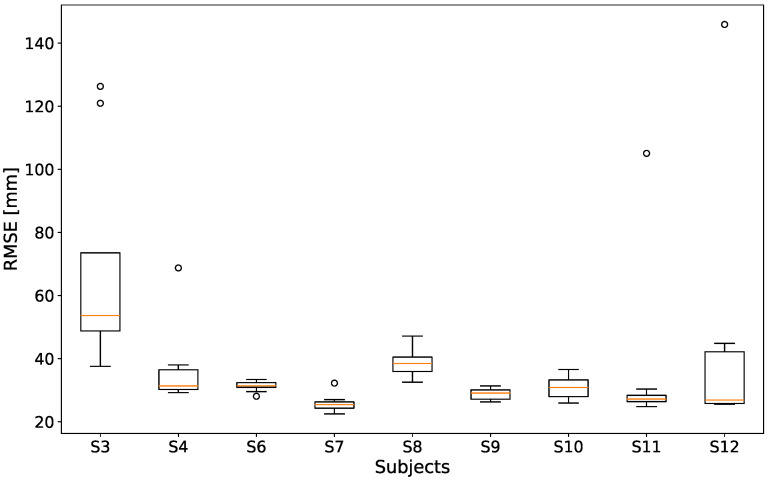
RMSE over all 81 recordings, for the unfiltered epipolar reconstruction, over the nine included subjects. For visual clarity, one singular outlier above 200 was excluded from the graph.

Figure 5 displays the RMSE for the unfiltered epipolar reconstruction method for each subject. Each individual boxplot contains nine videos, resulting in lower interquartile ranges. As can be seen, most subjects achieve a low RMSE (minimum median of 25.4 mm is achieved for subject 7), with the notable exception of subject 3, whose median RMSE value is as high as 53.6 mm.

Two notable things can be observed for subject 3 that can serve as explanations for the higher RMSE. Firstly, for the first trial, the lateral camera was wrongly positioned, so that the subject was partially outside of the cameras view. Secondly, from the lateral camera’s view, there is a jacket on a chair directly behind the subject. The jacket’s sleeve sometimes gets confused with the right arm, when the arm is obstructed by the body. The jacket is not present for any other subject. This again demonstrates one major limitation of our approach, as a noisy observation in one view deteriorates the overall estimation error, even though the second view detected the joint correctly.

**Figure 6:**
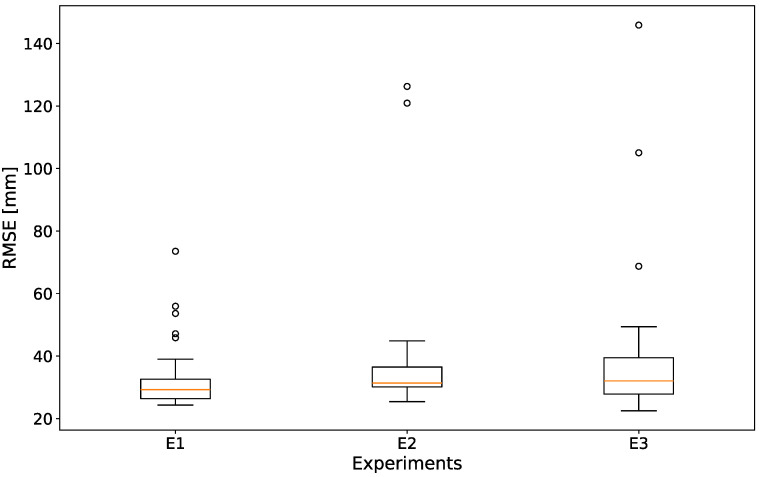
RMSE for all subjects, for the unfiltered epipolar reconstruction, over experiments.

Figure 6 shows the RMSE for the unfiltered epipolar method, for all subjects over the different experiments. Each boxplot contains 27 videos. As can be seen, no significant difference between the median values can be observed for the three experiments (E1: 29.2 mm, E2: 31.2 mm, E3: 32.0 mm, *p*-values of 0.270 and 0.200 between E1/E2 and E1/E3, respectively), with E3 having a slightly increased interquartile range. Thus, we can confidently state that the exercise quality does not have an influence on our reconstruction accuracy.

**Figure 7:**
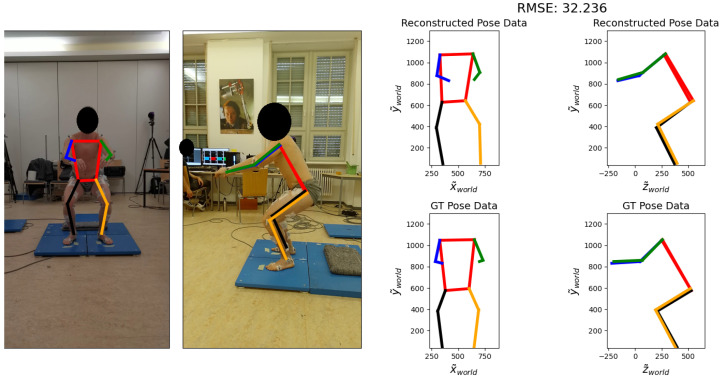
Visualization of the reconstruction for subject 7, experiment 1. On the left, frames from the frontal and lateral video are shown, with the MediaPipe 2D output drawn on top. On the right, the 2D projections onto the x-y-plane and y-z-plane of the reconstructed 3D pose (**top**) and the GT 3D pose (**bottom**) are shown.

Figure 7 shows an example visualization of the unfiltered epipolar reconstruction for a single frame taken from a recording of subject 7 during experiment 1. The RMSE over the whole recording is calculated as 32.2 mm.

### 3.2. Angle Accuracy

**Figure 8:**
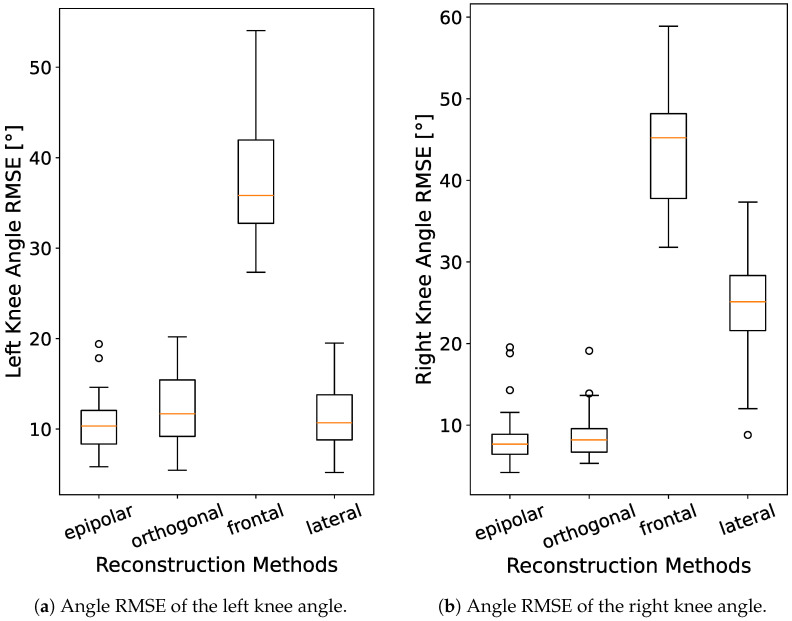
Angle RMSE of the left and right knee over all 81 recordings, for the different unfiltered reconstruction methods.

Figure 8a,b show the angle RMSE of the left and right knee angle, respectively. First of all, we notice the much higher RMSE for the 3D estimation based on the frontal camera. This is expected, since the angles are predominantly in the lateral plane. For the left side, we can see that the epipolar reconstruction achieves the lowest median of $10.3^{\circ }$, with the 3D MediaPipe data from the lateral camera being a close second at $10.7^{\circ }$ and the orthogonal reconstruction achieving slightly worse results than both. For the right side, however, the epipolar RMSE achieves an even lower median of $7.7^{\circ }$, while the lateral RMSE rises to $25.1^{\circ }$. This can easily be explained by the right side of the subject being obstructed by the left from a lateral view. Similar results can be seen when looking at the hip angle. The differences between the epipolar and orthogonal reconstruction are not significant (*p*-value of 0.070 and 0.695 for the left and right knee, respectively). The differences between epipolar reconstruction and the lateral single-camera approach are not significant for the left knee (p=0.375), but are significant for the right knee (p=0.000). When looking at the literature, Hancock et al. [34] have compared different goniometry methods used to measure knee angles. They found the digital inclinometer to be the most accurate method of assessment (6° minimum significant difference). The long arm goniometer had a minimum significant difference of 10°, while visual estimation and short arm goniometry were found to be equally inaccurate with 14° minimum significant difference. All of these measurements were made with a subject laying still on an operating table. In comparison, we can see that for our method, we can achieve comparable results with $10.7^{\circ }$ even though the subject was in motion.

**Figure 9:**
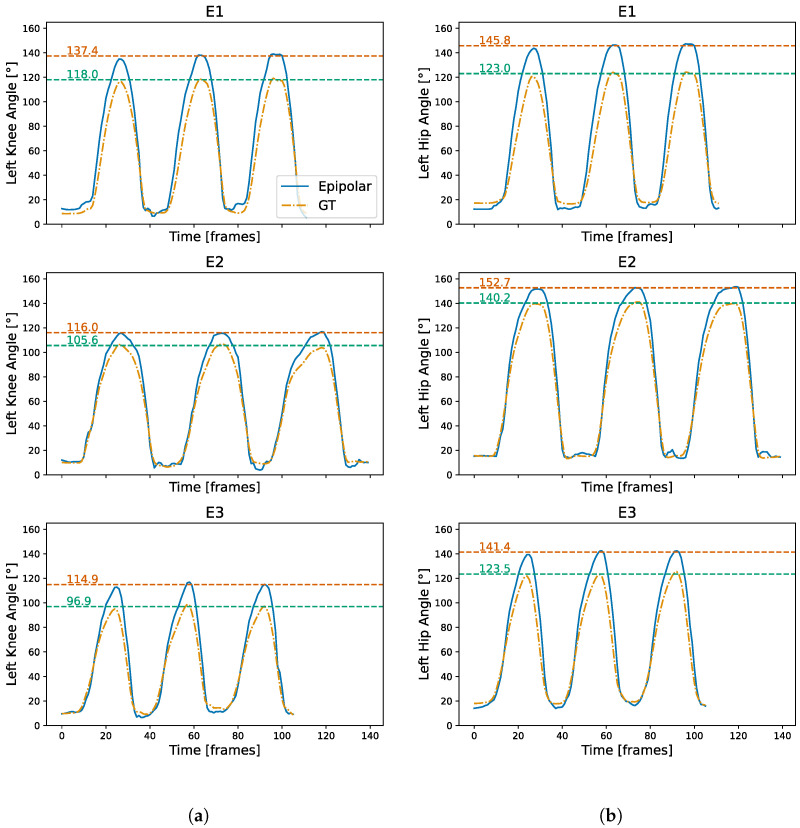
Example angle of the left knee (**left**) and hip (**right**) for the first three repetitions of the first sets of each experiment of subject 7. The red and green dotted lines show the mean value of the first three peaks for the unfiltered epipolar reconstruction and GT, respectively. (**a**) Left knee angle for E1 (**top**), E2 (**middle**) and E3 (**bottom**). (**b**) Left hip angle for E1 (**top**), E2 (**middle**) and E3 (**bottom**).

Figure 9 shows the absolute angle of the left knee and hip of subject 7 for the epipolar reconstruction and the GT. More specifically, Figure 9a shows the left knee angle of the first three repetitions of E1 (top), E2 (middle) and E3 (bottom), while Figure 9b shows the left for the three experiments. As can be seen, the reconstructed pose angle shows the same trends in the signal curve as the GT. The absolute error between the two curves is the highest when the angles reach their peak values, with the epipolar reconstruction consistently overestimating the maximum angle. However, despite these shortcomings of the reconstruction, we can still distinguish the three experiments from the mean peak values obtained from the epipolar reconstruction alone. The left knee angle can be used to separate the correct execution in E1 from the other two experiments, with a mean value of 137.4°, which is about 20° higher than the mean peak values for E2 and E3. Similarly, the left hip angle can be used to separate E3 from E2, with a mean peak value of 141.4°, which is more than 10° lower than the mean peak value for E2. These differences are very comparable to the values obtained from the GT.

## 4. Conclusions

In this work, we presented a method to employ MediaPipe Pose, epipolar geometry, and direct triangulation to reconstruct human 3D pose data from a stereo camera view. For this, we recorded a dataset featuring nine subjects performing squats. The subjects were recorded by two perpendicular cameras as well as a motion capture system. We used the 3D ground truth data provided by the motion capture system to analyze the estimation accuracy of our reconstructed 3D pose and compare our approach to a simpler fusion method as well as monocular 3D estimations. We analyzed both the residuals of a least-squares optimization between the reconstructed 3D pose data and the ground truth, which can be interpreted as the mean root-mean-square error over all joints and time, as well as the angle RMSE for the task-relevant knee angle. We could show that our approach outperforms monocular 3D estimation methods as well as straightforward orthogonal combination and achieves an RMSE of 30.9 mm, which is in line with current state-of-the-art approaches, while being more computationally efficient. We also showed that angles estimated on the reconstructed 3D data are as accurate as gold standard methods, with an RMSE of $10.7^{\circ }$. We could also show that the angles are an adequate metric to distinguish between a correct and incorrect squat. This indicates that our method is suitable for further usage in the analysis of physical exercises.

In a next step, we will validate our findings of a low RMSE of the reconstructed pose data for the task of physiotherapy analysis by classifying correct and incorrect exercises. Furthermore, we want to leverage the sensor data recorded in the dataset, namely electromyography and ground reaction force, to see if we can give an estimate for these based on our pose data. This would enable us to not only differentiate correct and incorrect exercises, but also make predictions about bio-mechanical processes happening during the exercise. In future works, we plan to make these predictions more reliable, by developing more sophisticated 3D reconstruction methods making use of bio-mechanical models, whose parameters are fitted through optimization and deconvolutional networks. This would also help alleviate the current problem where one erroneous observation deteriorates the reconstruction accuracy. Furthermore, we are working on extending the dataset both in sample size and exercise variety. Overall, this would mean an important step towards an automated evaluation system capable of quantitative and qualitative physical exercise evaluation.

## Figures and Tables

**Table 1:** Assignment of equivalent joint positions between the GT MoCap data and the MediaPipe joint labels. Each MediaPipe joint is assigned to either a single MoCap marker, a combination of MoCap markers, e.g., the mean of markers A6 and A8 for the left elbow, or a fictional marker created from the x-coordinate of A12 and the y-coordinate and z-coordinate of A16 for the left hip. Explanations for the marker names can be found in Table A1.

MoCap Joint Number	MediaPipe Marker Number	Joint Name
A4	B11	Left Shoulder
A5	B12	Right Shoulder
$\frac{A6+A8}{2}$	B13	Left Elbow
$\frac{A7+A9}{2}$	B14	Right Elbow
A14	B15	Left Wrist
A15	B16	Right Wrist
$\left[x_{A12},y_{A16},z_{A16}\right]$	B23	Left Hip
$\left[x_{A13},y_{A17},z_{A17}\right]$	B24	Right Hip
$\frac{A18+A20}{2}$	B25	Left Knee
$\frac{A19+A21}{2}$	B26	Right Knee
$\frac{A26+A28}{2}$	B27	Left Ankle
$\frac{A27+A2}{2}$	B28	Right Ankle

## Data Availability

The datasets presented in this article are not readily available because of privacy concerns.

## Abbreviations

The following abbreviations are used in this manuscript:

2D	Two-Dimensional
3D	Three-Dimensional
RMSE	Root Mean Square Error
MoCap	Motion Capture
RGB	red–green–blue
CMU	Carnegie Mellon University
CL	Lateral Camera
CF	Frontal Camera
IRC	Infrared Camera
GT	Ground Truth

## Appendix A

**Table A1:** Explanation of the MoCap marker numbers given in Figure 3a.

MoCap Joint Number	Anatomical Position
A1	Left Frontal Bone
A2	Right Frontal Bone
A3	Cervical Bone Processus 7 (Vertebra prominens)
A4	Left Acromion
A5	Right Acromion
A6	Left Anterior Elbow (caput commune of extensors)
A7	Right Anterior Elbow (caput commune of extensors)
A8	Left Posterior Elbow (caput commune of flexors)
A9	Right Posterior Elbow (caput commune of flexors)
A10	Left Posterior Spina Iliaca superior
A11	Right Posterior Spina Iliaca superior
A12	Left Anterior Spina Iliaca superior
A13	Right Anterior Spina Iliaca superior
A14	Left Wrist
A15	Right Wrist
A16	Left Hip (Trochanter Major)
A17	Right Hip (Trochanter Major)
A18	Left Lateral Knee (joint line)
A19	Right Lateral Knee (joint line)
A20	Left Medial Knee (joint line)
A21	Right Medial Knee (joint line)
A22	Left Metatarsal Joint 1
A23	Right Metatarsal Joint 1
A24	Left Metatarsal Joint 5
A25	Right Metatarsal Joint 5
A26	Left Lateral Ankle (Malleolus lateralis)
A27	Right Lateral Ankle (Malleolus lateralis)
A28	Left Medial Ankle
A29	Right Medial Ankle
A30	Left Heel (Tuber calcanei)
A31	Right Heel (Tuber calcanei)

**Table A2:** Explanation of the MediaPipe joint numbers given in Figure 3b.

MediaPipe Joint Number	Joint Explanation
B0	Nose
B1	Left Eye (inner)
B2	Left Eye
B3	Left Eye (outer)
B4	Right Eye (inner)
B5	Right Eye
B6	Right Eye (outer)
B7	Left Ear
B8	Right Ear
B9	Mouth (left)
B10	Mouth (right)
B11	Left Shoulder
B12	Right Shoulder
B13	Left Elbow
B14	Right Elbow
B15	Left Wrist
B16	Right Wrist
B17	Left Pinky
B18	Right Pinky
B19	Left Index
B20	Right Index
B21	Left Thumb
B22	Right Thumb
B23	Left Hip
B24	Right Hip
B25	Left Knee
B26	Right Knee
B27	Left Ankle
B28	Right Ankle
B29	Left Heel
B30	Right Heel
B31	Left Foot Index
B32	Right Foot Index
